# Three-Dimensional-Printed Meat Products with Lycopene-Functionalized Yeast Pickering Emulsions as Fat Replacer

**DOI:** 10.3390/foods14142518

**Published:** 2025-07-18

**Authors:** Zihan Cao, Yu Xing, Shasha Zhou, Feifan Li, Lixin Wang, Juanjuan Zhang, Xiaoxi Yang, Yumiao Lang

**Affiliations:** Key Laboratory of Public Health Safety of Hebei Province, College of Public Health, Hebei University, No. 180 Wusidong Road, Baoding 071002, China; caozihan616@163.com (Z.C.); cangan8882@yeah.net (Y.X.); zhoushasha1998@163.com (S.Z.); lifeifan23@163.com (F.L.); wanglixin0106@163.com (L.W.); zhangjuanjuan2425@163.com (J.Z.)

**Keywords:** lycopene, pickering emulsions, fat replacement, antioxidant properties, 3D printing

## Abstract

Due to the health-driven demand for fat replacers in meat products, Lycopene (Lyc)-loaded yeast protein (YP) high internal phase Pickering emulsions (HIPPEs) were explored as fat replacers for 3D-printed meat products. HIPPEs with varying Lyc concentrations were formulated, and their encapsulation efficiency and antioxidant activity (DPPH and ABTS assays) were evaluated. The encapsulation efficiency of Lyc exceeded 90% for all samples. Microscopic analysis revealed significant droplet enlargement in emulsions containing Lyc concentrations of 1.25 mg/mL and 1.50 mg/mL. Antioxidant activity peaked at a Lyc concentration of 1.00 mg/mL. Three-dimensional-printed meat products with different fat replacement ratios (0%, 25%, 50%, 75% and 100%) were prepared using both Lyc-loaded and non-loaded emulsions, and their printing precision, cooking loss, color, pH, texture, and lipid oxidation were assessed. The replacement ratio had no significant impact on printing precision, while cooking yield improved with higher fat replacement levels. Lyc emulsions notably influenced meat color, resulting in lower lightness and higher redness and yellowness. pH values remained stable across formulations. Lipid oxidation decreased with increasing fat replacement levels. The results indicate that Lyc-loaded YP Pickering emulsions have great potential as effective fat replacers for 3D-printed meat products, enhancing antioxidant performance while preserving product quality.

## 1. Introduction

The rapid growth of the global economy and improvements in living standards have heightened public awareness of food nutrition and health. Among various food categories, meat products remain popular due to their rich nutritional composition, including proteins, fats, vitamins, and minerals, as well as their desirable taste and texture [1,2,3]. However, the high levels of saturated fatty acids and trans fatty acids in conventional meat products are linked to adverse health effects, such as cardiovascular diseases, type 2 diabetes, and obesity [2,4]. The World Health Organization (WHO) reports that over 2.5 billion people are overweight, with more than 890 million classified as obese, underscoring the urgent need for healthier dietary alternatives [5]. Efforts to develop healthier and more sustainable meat products often face the challenge of balancing nutritional benefits with sensory quality. Some advanced technologies, including the integration of sophisticated processing and formulation strategies—such as utilizing high-moisture extrusion to develop fibrous textures [6], enhancing flavor and juiciness through aging [7], and employing structured lipid systems (like oleogels and Pickering emulsions) to replicate the mouthfeel of fat—can render meat products both healthier and more sensorially appealing [8]. While reducing fat content in formulations can mitigate health risks, it often leads to impaired taste, texture, and consumer acceptability. Substituting animal fats with plant-based oils has been widely explored, particularly with oils like canola [9] and olive oil [10], which offer promising health benefits. Canola oil, one of the most widely consumed edible oils, is notable for its high content of polyunsaturated fatty acids (PUFAs), including omega-6 and omega-3 fatty acids, and its relatively low levels of saturated fatty acids [11]. However, the incorporation of vegetable oils presents challenges in preserving the sensory and structural properties essential for meat products. This situation necessitates the development of innovative fat replacement strategies, including the production of structured vegetable oil gels (oleogels) [12], pre-emulsified oil systems, elastic gel networks [12,13], and Pickering emulsions.

Pickering emulsions, stabilized by solid particles rather than traditional surfactants, have emerged as an effective strategy for addressing these challenges [14]. These food-grade emulsions exhibit exceptional stability, environmental friendliness, and adaptability, making them highly suitable for various applications, including meat product formulation. HIPPEs are typically characterized by an exceptionally high dispersed phase volume fraction that exceeds 74% [15]. These systems generally form densely packed networks of oil droplets stabilized by solid particles at the interface, eliminating the need for synthetic surfactants [16]. HIPPEs exhibit solid-like properties, distinctive rheology, and stability at high internal phase volumes, making them well-suited for delivering hydrophobic bioactives and for use in 3D printing applications [17]. For example, HIPPEs stabilized by citrus pectin–β-cyclodextrin have been successfully utilized as printable inks [18]. Additionally, protein–cellulose HIPPE gels facilitate the extrusion printing of structures that mimic fat networks. Moreover, dried HIPPE templates serve as bioactive scaffolds for tissue engineering applications [19]. Keum et al. [20] investigated the physicochemical properties of polysaccharide/pea protein complex Pickering emulsions and their application in plant-based meat products, demonstrating that a partial replacement with methylcellulose and pea protein isolate can effectively substitute plant oils in plant-based meat products. Rezaee [21] studied novel Pickering emulsions stabilized by double-low canola protein microgels and xanthan gum, evaluating their potential as animal fat substitutes in meat systems. Their research indicates that it is feasible to develop fat substitutes for meat systems that possess enhanced stability, improved nutritional profiles, and greater oxidative stability.

However, high-fat meat products are prone to oxidation due to their chemical structure, which may lead to adverse sensory and nutritional changes [22]. Plant oils in Pickering emulsions, owing to their high unsaturated fatty acid content, are particularly susceptible to oxidation [23]. Therefore, incorporating antioxidants is crucial, especially in fat-substitutes meat products. Lyc is a naturally occurring carotenoid with strong antioxidant activity, mainly found in tomatoes, watermelon, and grapefruit. In addition to its antioxidant capacity, Lyc has been recognized for its physiological functionality, including anti-inflammatory, anti-cancer, and anti-apoptotic effects [24]. Whey protein fibril–alginate stabilized Pickering emulsions have been effectively utilized to encapsulate Lyc, thereby enhancing its stability and bioaccessibility under thermal and digestive conditions [25]. HIPPEs loaded with Lyc, prepared using ovalbumin–chitosan complexes, demonstrate robust structural integrity and show promise for applications in customizable food inks [26]. These studies have demonstrated the potential of Pickering emulsions in functionalizing yeast-based systems with Lyc.

Meanwhile, advancements in 3D food printing technology provide a transformative platform for producing personalized and customized food products. Through layer-by-layer deposition of food materials, this technology enables precise control over shape, texture, nutritional content, and aesthetic appeal [27,28,29,30,31]. Three-dimensional printing has garnered significant attention due to its advantages, such as flexibility, customizable nutrition profiles, and labor-saving processes [32]. When combined with innovative fat substitutes like Pickering emulsions, 3D printing technology can further enhance the sensory and functional properties of meat products. Additionally, 3D-printed minced meat is easier to chew and digest, offering a novel solution to improve nutritional intake for older adults and patients [32]. Moreover, 3D printing facilitates the development of personalized and custom-designed foods, including products with intricate shapes and complex geometries, making them particularly appealing to target groups such as children and adolescents [33]. Although protein-stabilized Pickering emulsions and Lyc fortification have been studied separately, their combination in 3D-printed meat products is novel.

This study investigates the incorporation of Lyc into YP-stabilized Pickering emulsions for application in 3D-printed meat products. By harnessing the synergistic benefits of Pickering emulsions, 3D printing, and Lyc, this research aims to develop an innovative fat replacement strategy that reduces fat content, enhances product quality, and improves health outcomes. This research offers innovative strategies for the 3D printing of low-fat meat products, thereby promoting consumer health through the development of functional meat alternatives.

## 2. Materials and Methods

### 2.1. Materials

Pork was sourced from Baoding Lianchi Meat Joint Processing Factory (Baoding, China). Canola oil was purchased from Yihai Kerry Arawana Holdings Co., Ltd. (Shanghai, China). Yeast powder (total nitrogen: ≥10.0%; amino nitrogen: ≥4.0%; moisture: ≤6.0%; sodium chloride: ≤2.0%; ash: ≤15.0%) was obtained from Angel Yeast (Yichang, China). Lyc (≥98% purity) was purchased from Aladdin (Shanghai, China). All chemicals were of analytical grade and used without further purification. Distilled water was used in all experiments.

### 2.2. YP Extraction

YP was extracted based on the method previously reported by Xia et al. [34] with slight modifications. Yeast powder was combined with five volumes of yeast lysis buffer (1% (*w*/*v*) sodium dodecyl sulfate (SDS), 5% (*w*/*v*) sodium bisulfite (NaHSO_3_), and 1.8% (*w*/*v*) sodium hydroxide (NaOH)) and stirred thoroughly. The mixture was then incubated in a water bath at 85 °C for 1 h with intermittent homogenization. After heat treatment, the mixture was cooled to room temperature, and the pH was adjusted to 12.0 using sodium hydroxide (NaOH, 2.5 mol/L). The mixture was then centrifuged at 5000 rpm for 10 min at 4 °C. The precipitate was collected and mixed with an equal volume of lysate, and the heating and centrifugation steps were repeated. The supernatant was then adjusted to pH 4.2 to precipitate the dissolved proteins. After centrifugation at 5000 rpm for 10 min at 4 °C, the supernatant was discarded, and the precipitate was collected as YP extract.

### 2.3. Preparation of Lyc-Loaded HIPPEs

A specified amount of Lyc was dissolved in canola oil to achieve concentrations of 0.50, 0.75, 1.00, 1.25, and 1.50 mg/mL. The solutions were stirred until Lyc was completely dissolved, resulting in Lyc-loaded oil phases. A 3.00 mL solution of YP (2.50 mg/mL, pH 5.5) was used as the aqueous phase, and 17.00 mL of canola oil with varying Lyc concentrations was used as the oil phase. The mixture was then homogenized using a high-speed shear emulsifier (XHF-DY, Ningbo Scientz Biotechnology Co., Ningbo, China) at 13,000 rpm for two 30 s cycles to obtain Lyc-loaded YP-stabilized HIPPEs.

### 2.4. Characteristics of Lyc-Stabilized HIPPEs

#### 2.4.1. Encapsulation of Lyc in HIPPEs

The encapsulation efficiency of Lyc in the emulsions was calculated using a UV-Vis spectrophotometer (UV-2550, Shimadzu, Kyoto, Japan), following the method described by Lv et al. [35]. An aliquot of 0.20 mL of Lyc-loaded YP-stabilized HIPPEs was dispersed in a 3 mL mixture of anhydrous ethanol and n-hexane (1:2, *v*/*v*), centrifuged at 2320× *g* for 10 min, and the supernatant was collected. This process was repeated three times. Using n-hexane as the blank, the absorbance was measured at 471 nm. Lyc concentration was calculated based on a standard calibration curve constructed from serial dilutions of a Lyc standard solution. The calibration equation was A = 0.3582C + 0.0028, with a linear range of 0.16–1.76 μg/mL and R^2^ = 0.9937. Encapsulation efficiency (EE) was calculated using Equation (1):

(1) $$\mathrm{EE}=\frac{C_{1}-C_{0}}{C_{0}}\;\times \;100\%$$

where C_1_ is the concentration of Lyc in the fresh emulsion (mg/mL), and C_0_ is the initial concentration of Lyc added before homogenization (mg/mL).

#### 2.4.2. Microscopic Structure Evaluation

A 20 µL aliquot of the homogenized emulsion was placed on a glass slide, covered with a coverslip to ensure no air bubbles, and examined and photographed under an optical microscope (ECLIPSE Ts2, Nikon Co., Kyoto, Japan) equipped with 10× and 40× objective lenses.

#### 2.4.3. Color Measurement

Color measurements of the emulsions were performed using a spectrophotometer (3NH YS3010, Shenzhen Threenh Technology Co., Shenzhen, China) as described by Cen et al. [36]. The L* (lightness), a* (redness/greenness), and b* (yellowness/blueness) values were recorded. The whiteness (W) values were calculated using Equation (2):

(2) $$W=100-\sqrt{{\left(100-L^{*}\right)}^{2}+{\left(a^{*}\right)}^{2}+{\left(b^{*}\right)}^{2}}$$

#### 2.4.4. Antioxidant Capacity

The antioxidant capacity of the emulsions can be evaluated by DPPH and ABTS radical scavenging assays. DPPH (4.00 mg) was dissolved in ethanol and diluted to a final volume of 100 mL to prepare a 0.10 mmol/L DPPH stock solution [36]. A 20 µL aliquot of the sample solution was mixed with 3.00 mL of the DPPH stock solution in a test tube, shaken, and allowed to react in the dark at room temperature for 30 min. The absorbance at 517 nm was measured (*A*_1_). The background absorbance of the sample solution was recorded using ethanol instead of DPPH (*A*_2_), and the blank absorbance was measured with ethanol + DPPH (*A*_0_). The DPPH radical scavenging activity was calculated using Equation (3):

(3) $$DPPH\;Radical\;Scavenging\;Rate\;\left(\%\right)=\frac{A_{1}-A_{2}}{A_{0}}\times 100\%$$

ABTS radical scavenging activity was determined according to Smith et al. [37] with minor modifications. Briefly, an ABTS solution (7 mmol/L) was mixed with a potassium persulfate solution (2.45 mmol/L) in a 1:1 ratio and stored in the dark at room temperature for 12–16 h. The solution was then diluted with ethanol to achieve an absorbance of 0.70 ± 0.02 at 734 nm. A 3 mL aliquot of this solution was mixed with 20 μL of the emulsion and allowed to stand for 6 min. The absorbance at 734 nm was measured (*A*_1_). The background absorbance of the sample solution was recorded using ethanol instead of ABTS (*A*_2_), and the blank absorbance was measured with ethanol + ABTS (*A*_0_). The ABTS radical scavenging activity was calculated using Equation (4):

(4) $$ABTS\;Radical\;Scavenging\;Rate\;\left(\%\right)=\frac{A_{1}-A_{2}}{A_{0}}\times 100\%$$

### 2.5. Characteristics of 3D-Printed Meat

#### 2.5.1. Meat Preparation and 3D Printing

Five pork formulations were prepared following methods described in previous literature with slight modifications as shown in Table 1 [37]. The control formulation consisted of fresh lean meat (80%, *w*/*w*) and ground pork fat (20%, *w*/*w*). In modified groups, Lyc-loaded or non-loaded emulsions were used to replace pork fat in varying proportions (0%, 25%, 50%, 75%, and 100%).

#### 2.5.2. Printing Accuracy

Printing accuracy was evaluated by calculating the deviation between the designed dimensions (length, width, height) and the actual dimensions of the printed blocks [29]. After stabilization, the dimensions were measured using a digital caliper (LY2919059, Shenzhen Luxianzi Technology Co., Ltd., Shenzhen, China) and compared to the model specifications. The printing accuracy equation was determined according to the method of John Robert Honiball et al. [38]. The accuracy was calculated via Equation (5). The results were averaged from triplicate trials. Additionally, the printed blocks were weighed, photographed, and documented where necessary.

(5) $$Printing\;accuracy\;(\%)=\frac{V_{2}}{V_{1}}\times 100\%$$

where *V*_1_ is the design volume of the meat product; *V*_2_ is the actual volume of the meat product.

#### 2.5.3. Cooking Yield

Before cooking, the meat samples were weighed to obtain *m*_1_. After cooking, they were allowed to cool to room temperature for 30 min, and the surface moisture was blotted with filter paper and reweighed (*m*_2_) [39]. The cooking yield was calculated via Equation (6). Three sets of replicate experiments were performed for each sample.

(6) $$Cooking\;yield\;(\%)=\frac{m_{2}}{m_{1}}\times 100\%$$

#### 2.5.4. Color

The changes in the appearance of meat products before and after cooking, as well as differences among samples with varying fat replacement ratios, were observed. Color measurements of cooked meat were performed using a colorimeter (3NH YS3010, Shenzhen 3NH Technology Co., Ltd., Shenzhen, China), recording L*, a*, and b* values [21]. The meat was sliced to a 5 mm thickness, ensuring coverage of the instrument’s probe, and measurements were taken using D65 lighting(YS3010, Shenzhen Sanen Times Technology Co., Ltd., Shenzhen, China) and a 10° angle.

#### 2.5.5. pH Analysis

Following the method of Arun et al. with slight modifications [40]. Two grams of cooked meat samples was homogenized in 20 mL of 0.1 mol/L potassium chloride solution, and the pH was measured (PHS-3BW, Shanghai Bante Instrument Co., Ltd., Shanghai, China).

#### 2.5.6. Lipid Oxidation

The TBARS method was modified according to Li et al. [41] to determine the level of malondialdehyde (MDA). Briefly, 20 mL of a solution containing 0.1% (*w*/*v*) EDTA and 7.5% (*w*/*v*) TCA was mixed with 2 g of the meat sample and incubated in a 50 °C water bath for 30 min with constant shaking. After cooling to room temperature, the mixture was filtered. An aliquot of 1 mL of the filtrate was combined with 1 mL of TBA solution and heated at 90 °C for 30 min. The absorbance of the solution was measured at 532 nm using a UV-Vis spectrophotometer (UV-2550, Shimadzu, Nikon, Tokyo, Japan). The concentration of MDA in the meat samples was determined using a standard curve based on 1,1,3,3-tetraethoxypropane. The MDA content in the emulsion was calculated using Equation (6):

(7) $$X=\frac{c\times v\times 1000}{m\times 1000}$$

where *X* is the content of malondialdehyde in the emulsion (mg/kg), *c* is the concentration of malondialdehyde in the emulsion (µg/mL), *v* is the constant volume of the sample solution (mL), *m* is the sample mass represented by the final sample solution (g), and 1000 is the conversion coefficient.

#### 2.5.7. Texture Profile Analysis

Texture profile analysis (TPA) was performed with modifications based on the method described by Zhuang et al. [42]. Cooked meat samples (1 cm × 1 cm × 1 cm) were analyzed for texture characteristics using a texture analyzer (TA.XTC-18, Shanghai Baosheng Industrial Development Co., Ltd., Shanghai, China). The parameters were set as follows: probe model TA/36, compression distance of 5 mm, trigger force of 5 g, pre-test speed of 2.0 mm/s, and test speed of 0.5 mm/s. The hardness, chewiness, elasticity, cohesiveness, and resilience of the meat samples were measured.

### 2.6. Statistical Analysis

Statistical analysis was performed using IBM SPSS Statistics 27 (IBM Corp., Armonk, NY, USA). One-way ANOVA followed by Tukey’s HSD test was used to determine significant differences (*p* < 0.05). Independent-samples *t*-tests were applied to compare Lyc-loaded and non-loaded groups. The results are expressed as mean ± standard deviation (*n* = 3).

## 3. Results and Discussion

### 3.1. Characterization of Lyc-Stabilized HIPPEs

#### 3.1.1. Encapsulation of Lyc in HIPPEs

**Figure 1:**
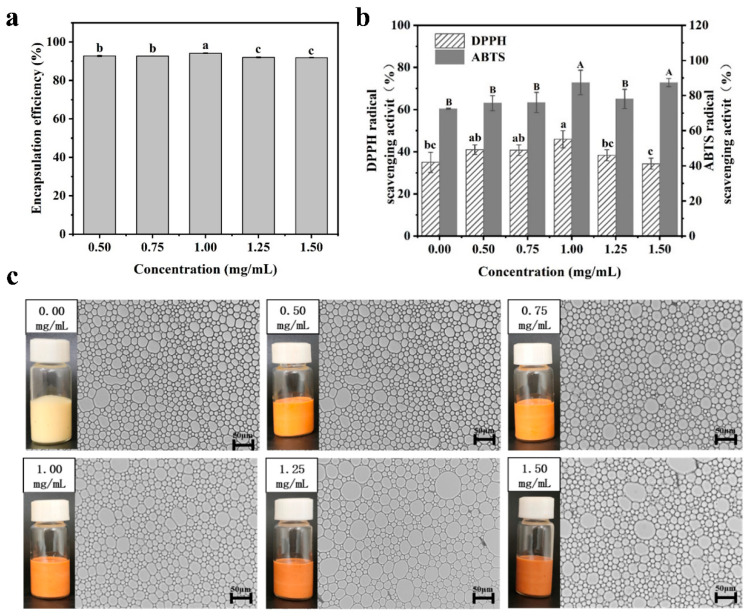
Effect of Lyc concentration on EE (**a**); effect of Lyc concentration on antioxidant properties of emulsions (**b**); microstructure (400×) and appearance of different Lyc emulsions (**c**). Different uppercase letters indicate significant differences among ABTS values (*p* < 0.05), while different lowercase letters indicate significant differences among DPPH values (*p* < 0.05).

The encapsulation efficiency of Lyc remained consistently above 90% (*p* < 0.05, Figure 1a), primarily due to its strong compatibility with the lipid phase in the Pickering emulsion system. The high oil solubility of Lyc (logP = 17.64, Zahari et al. [43]) facilitates its rapid partitioning into the hydrophobic cores of YP-stabilized oil droplets. YP, with its amphiphilic nature, forms a compact interfacial film around oil droplets [44]. Oil-in-water (*O*/*W*) emulsions are particularly suitable for dissolving lipophilic compounds like Lyc, enhancing their stability and bioavailability, thus playing a key role in encapsulation [45]. This behavior is consistent with findings from other lipid-based encapsulation systems. For example, Zein, a plant-derived amphiphilic protein, has been shown to self-assemble into interfacial films that effectively entrap lipophilic compounds through hydrophobic interactions, enhancing encapsulation efficiency and stability [46].

#### 3.1.2. Antioxidant Ability

The DPPH and ABTS methods are commonly used to determine radical content and assess antioxidant activity [47,48]. The conjugated double-bond system of Lyc has strong singlet oxygen quenching capability, making it a common natural antioxidant in food science [49]. As shown in Figure 1b, different concentrations of Lyc achieved over 35% scavenging rate for DPPH radicals, with the highest scavenging rates for DPPH radicals, with the highest scavenging rates for both DPPH and ABTS radicals observed at a concentration of 1.00 mg/mL (*p* < 0.05). This is consistent with previous findings that the encapsulation efficiency of Lyc decreases at higher concentrations. Lyc itself is a potent antioxidant, and increasing its concentration enhances its radical-scavenging capacity. However, when the concentration exceeded a certain level, the scavenging efficiency plateaued or slightly declined. A similar concentration-dependent trend has been reported by Sen Gupta et al. [50], indicating that the ABTS activity of α- and β-Carotene isolated from Crude Palm Oil reaches its peak at a concentration of 0.05%. Furthermore, the DPPH of α- and β-Carotene was highest at low concentrations. These variations may be attributed to the inherent color properties of Lyc or carotenoids, which can interfere with measurement accuracy and result in diminished DPPH activity at elevated concentrations [50]. Additionally, excessively high concentrations of antioxidant compounds may induce pro-oxidative effects by inhibiting oxidation systems, consequently leading to oxidative stress [50].

#### 3.1.3. Morphology Evaluation

Microscopy images of freshly prepared YP-stabilized HIPPEs loaded with Lyc are shown in Figure 1c. The images reveal that the droplets of all Lyc-loaded YP-stabilized HIPPEs exhibit a spherical morphology. At Lyc concentrations of 1.25 mg/mL and 1.50 mg/mL, the droplets appeared visually larger than those at lower concentrations or without Lyc, indicating that increased Lyc levels may affect the droplet morphology of HIPPEs. Mechanistically, hydrophobic Lyc is likely to adsorb onto yeast particles at the oil–water interface, therefore reducing particle wettability and diminishing the rigidity of the interfacial film [51]. This phenomenon results in looser particle packing, which contributes to droplet enlargement while still preventing coalescence through steric hindrance [52]. Similar behavior has been observed in other Pickering systems, for example, emulsions stabilized by tannic acid/starch that are loaded with Lyc maintain droplet integrity but exhibit an increase in size due to thicker yet less compact interfacial layers [53,54].

#### 3.1.4. Color

The color measurements confirmed that increasing Lyc content strongly affected the emulsion color. The lightness (L*), yellowness (b*), and whiteness (W) values of the emulsions significantly decreased with increasing Lyc concentration (*p* < 0.05), whereas redness (a*) increased (*p* < 0.05). The emulsions became more intensely orange as the Lyc concentration increased, which is consistent with the appearance of HIPPEs shown in Table 2. These color changes are attributed to the spectral properties of Lyc, which is a deeply colored carotenoid. Thus, higher concentrations absorb more incident light [55,56], causing the emulsion to lose brightness. The increase in a* and decline in b* indicates that the added red pigment dominates the color, reducing the relative yellow component.

### 3.2. Characteristics of 3D-Printed Meat

#### 3.2.1. Printing Accuracy

**Figure 2:**
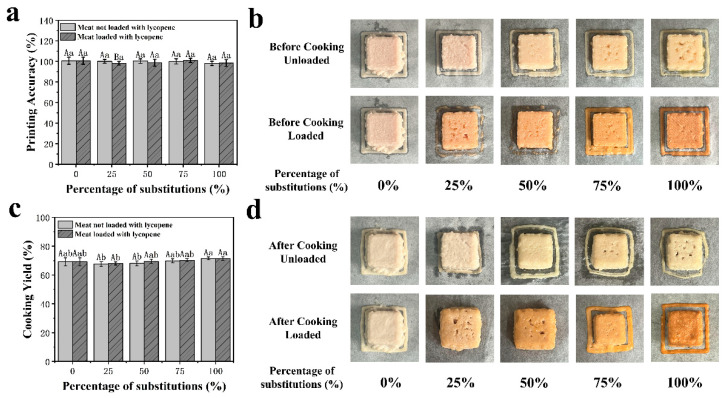
Printing accuracy (**a**) and appearance (**b**) of meat products printed with different proportions of emulsions replacing fat. Cooking yield (**c**) and appearance (**d**) of cooked meat products prepared with different proportions of emulsions replacing fat. ^a,b^ Different letters indicate significant differences among substitution levels within the Lyc-loaded group (*p* < 0.05). ^A,B^ Different letters indicate significant differences between Lyc-loaded and non-loaded samples at the same substitution level (*p* < 0.05). Values are expressed as mean ± standard deviation (SD).

Printing accuracy is crucial for maintaining the nutritional integrity, texture, shape, and stability of 3D-printed foods. In this study, the printing accuracy, defined as the ratio of measured to set volume, remained near 100%, with a mean of 99.55% ± 2.50% and a range of 98.12% to 100.99%. Values slightly exceeding 100% are commonly observed when printing accuracy is calculated based on volume ratio, and are typically attributed to over-extrusion or slight material expansion during deposition [57]. No significant differences were observed across fat substitution ratios (*p* > 0.05, Figure 2a), indicating that the substitution levels did not affect dimensional accuracy. This stability suggests that the controlled deposition of the emulsion within the predefined sample boundaries prevented structural deformation caused by fat leakage. These findings align with those of Dick et al. [29], who reported that fat replacement strategies did not compromise the morphological accuracy of 3D-printed beef.

#### 3.2.2. Cooking Loss

Cooking loss is a critical parameter reflecting the water-holding capacity (WHC) of meat products during processing, directly influencing their texture, quality, and consumer acceptability. The cooking performance results are presented in Figure 2d. As shown, the incorporation of Lyc does not exert a statistically significant impact on the cooking yield of meat products (*p* > 0.05; Figure 2c). However, an increasing trend in cooking yield is observed with higher substitution ratios. This enhancement likely stems from the dense network structure formed in emulsified meat products, which improves their water-binding ability. Such a structure minimizes moisture and nutrient loss during thermal processing, thereby reducing cooking loss and enhancing overall product quality [58]. These findings highlight the potential of incorporating Lyc-loaded emulsions as functional fat replacers in meat formulations, contributing to improved processing performance and product stability without compromising textural or sensory properties.

#### 3.2.3. Color Analysis

Color parameters are key determinants in consumer purchasing decisions, significantly influencing the market performance of meat products. Varying substitution ratios significantly affect the color attributes of meat products (*p* < 0.05; Table 3).

The L* value decreased significantly in Lyc-loaded emulsion substitution groups (E1 to E4), with E4 showing a value of 64.92 ± 0.95. This trend can be attributed to the intrinsic color properties of Lyc, which has strong absorption due to its eleven linear conjugated double-bond structure [59]. As Lyc concentration increases, more light is absorbed in this region, resulting in lower L* values.

The a* value, indicative of red-green chromaticity, increased with higher fat substitution ratios in both Lyc-loaded and non-loaded emulsion groups, signifying enhanced redness of the meat products. The b* value, representing yellow–blue chromaticity, also increased significantly with higher substitution ratios, with a more pronounced effect observed in Lyc-loaded emulsions. García (2009) [60] reported similar results with dried tomato skins in beef burgers containing Lyc. Although Lyc is a red pigment, its addition to meat induces a color shift towards orange, which may explain the increased a* and b* parameters in Lyc-containing batches.

#### 3.2.4. pH Analysis

**Figure 3:**
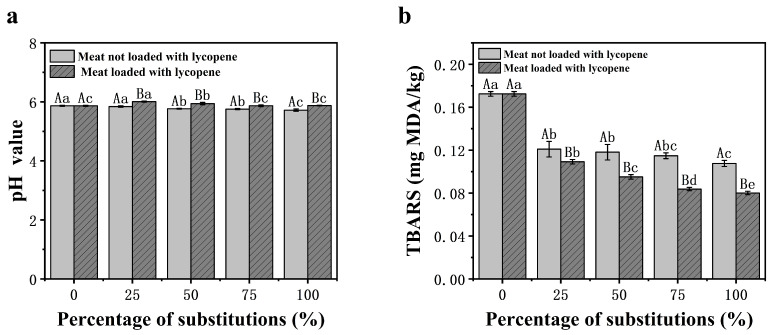
pH values (**a**) and lipid oxidation (**b**) of meat products printed with different proportions of emulsions replacing fat. ^a–e^ Different letters indicate significant differences among substitution levels within the Lyc-loaded group (*p* < 0.05). ^A,B^ Different letters indicate significant differences between Lyc-loaded and non-loaded samples at the same substitution level (*p* < 0.05). Values are expressed as mean ± standard deviation (SD).

pH plays a vital role in determining the functional properties of meat systems, including emulsification, gelation, and WHC. Monitoring pH levels is therefore critical in evaluating the quality and stability of meat products. The pH results for the two groups, as presented in Figure 3a, demonstrate no significant differences across substitution ratios (*p* > 0.05). This indicates that the incorporation of Lyc-loaded emulsions as fat substitutes does not influence the pH of the meat products. These findings align with the study by Rezaee et al. [21], which also reported no significant pH changes when double-low canola protein microgels and xanthan gum-stabilized Pickering emulsions were used as fat replacers.

#### 3.2.5. Lipid Oxidation

The thiobarbituric acid reactive substances (TBARS) value quantifies secondary oxidation products, such as malondialdehyde, in meat, providing an indicator of lipid oxidation levels. Lipid oxidation impacts meat color, texture, and flavor, serving as the primary driver of quality deterioration in meat and meat products [61]. TBARS values in both Lyc-loaded and non-loaded groups decreased significantly (*p* < 0.05) with increasing fat replacement ratios (25–100%) compared to the control group (T1, 0% substitution; Figure 3b). Notably, the Lyc-loaded groups showed a more pronounced reduction, suggesting that Lyc contributed to enhanced oxidative stability.

This antioxidant effect can be attributed to two primary mechanisms. First, Lyc, a potent carotenoid, exhibits superior singlet oxygen quenching due to its conjugated diene structure, along with strong metal chelation capabilities [49,62]. Among naturally occurring carotenoids, Lyc is regarded as one of the most effective antioxidants, primarily due to its highly unsaturated molecular backbone, which enhances its free radical scavenging capacity. Second, to further protect Lyc from oxidative degradation, canola oil was encapsulated within YP-stabilized Pickering emulsions, minimizing oxygen exposure and reducing the susceptibility of Lyc to oxidation. As a result, lipid oxidation was effectively suppressed in both the emulsion matrix and the final meat products (*p* < 0.05 vs. control; Figure 3b). These findings suggest that Lyc-loaded YP Pickering emulsions not only serve as a viable fat replacement strategy but also significantly enhance the oxidative stability of 3D-printed meat products, potentially extending their shelf life and improving overall product quality.

#### 3.2.6. Texture Profile Analysis

Fat significantly influences the textural properties of meat. Changes in fat content may have a considerable impact on meat texture. Texture measurements for various ratios of meat products are shown in Table 4. The elasticity, resilience, and cohesiveness of both groups are similar to the control group (*p* > 0.05), while chewiness and hardness were significantly higher than those of the control group, with values increasing progressively with higher substitution ratios (*p* < 0.05). Previous research [63] found that emulsions made from olive oil and alginate to replace sausage fat resulted in higher hardness and chewiness compared to control samples, consistent with this study’s findings. The increase in hardness and chewiness of meat products with higher substitution ratios may be attributed to the unique properties of canola oil used in emulsion preparation. The high unsaturated fatty acid content of canola oil, along with its emulsifying properties, influences the protein–fat matrix to form a more structured and cohesive gel system, thereby increasing the hardness and chewiness of the meat [57,64,65,66]. Additionally, the stability of Pickering emulsions can reduce water loss during processing, further contributing to the enhancement of textural properties. Hardness and chewiness are generally positively correlated; greater hardness leads to increased chewing resistance, thereby elevating chewiness. This result is consistent with Choi’s study [67], which found that plant oil-based emulsions increased meat hardness. There were no significant changes in cohesiveness, resilience, or elasticity compared to the control group, indicating that fat substitution with emulsions is feasible.

## 4. Conclusions

This study demonstrates the potential of Lyc-loaded YP Pickering emulsions as effective fat replacers in 3D-printed meat products, contributing to the development of healthier, functional alternatives. The incorporation of Lyc significantly improved the oxidative stability of the emulsions and the final products, effectively inhibiting lipid oxidation without compromising key physicochemical properties. Fat substitution not only preserved printing accuracy, pH, and cooking yield but also enhanced color characteristics (a*) and yellowness (b*), leading to improved visual appeal.

These findings highlight the dual functionality of Lyc-loaded Pickering emulsions as both fat replacers and natural antioxidants, offering a promising strategy for enhancing the stability and sensory attributes of 3D-printed meat products. Future research should explore optimizing formulation parameters, assessing long-term stability, and evaluating consumer acceptance to facilitate broader applications in meat product development.

## Figures and Tables

**Table 1:** Meat formulation with Lyc-loaded YP Pickering emulsion as a substitute for pork back fat.

Ingredient (g)	T1	T2	T3	T4	T5
Lean pork	24.00	24.00	24.00	24.00	24.00
Salt	0.80	0.80	0.80	0.80	0.80
Pork back fat	8.00	6.00	4.00	2.00	0.00
Emulsion (φ = 0.85)	0.00	2.35	4.71	7.06	9.41
Water	7.20	6.85	6.49	6.14	5.79
Total	40.00	40.00	40.00	40.00	40.00

Note: T1, T2, T3, T4, and T5 represent 0%, 25%, 50%, 75%, and 100% pork back-fat substitution by Lyc-loaded YP Pickering emulsion, respectively. The formulations were printed using a 3D printer (Wiiboox Sweetin, Nanjing Wiiboox 3D Technology Co., Ltd., Nanjing, China). The composite meat products were loaded into a syringe and printed into solid rectangular blocks (20 mm × 20 mm × 10 mm) to evaluate printing accuracy. The printing parameters included: printing precision: 0.2 mm, printing speed: 25 mm/s, nozzle diameter: 0.84 mm, platform height: −2 mm, and nozzle temperature: 25 °C. These parameters were determined through preliminary experiments. The printing program was then initiated.

**Table 2:** Effect of Lyc concentrations on the color.

Lyc Concentration (mg/mL)	L*	a*	b*	W
0.00	81.15 ± 0.08 ^a^	−4.27 ± 0.07 ^b^	29.52 ± 0.03 ^a^	64.72 ± 0.06 ^a^
0.50	62.55 ± 0.79 ^b^	17.54 ± 0.78 ^a^	27.96 ± 1.77 ^ab^	50.06 ± 0.68 ^b^
0.75	61.24 ± 1.13 ^bc^	17.92 ± 0.91 ^a^	27.29 ± 0.64 ^b^	49.32 ± 1.53 ^b^
1.00	60.34 ± 0.31 ^cd^	18.52 ± 0.80 ^a^	26.56 ± 0.55 ^bc^	48.80 ± 0.81 ^b^
1.25	58.63 ± 0.04 ^de^	18.06 ± 0.92 ^a^	24.98 ± 0.65 ^cd^	48.41 ± 0.67 ^b^
1.50	57.66 ± 1.48 ^e^	18.44 ± 0.10 ^a^	24.30 ± 0.22 ^d^	47.81 ± 1.14 ^b^

Note: L*: lightness; a*: redness/greenness; b*: yellowness/blueness; W: whiteness values. ^a–e^ Different letters indicate significant difference in the same column (*p* < 0.05). Values are expressed as mean ± standard deviation (SD).

**Table 3:** Color parameters of meat products printed with different proportions of emulsions replacing fat.

Parameters	L*	a*	b*
blank	78.36 ± 1.54 ^Aab^	0.50 ± 0.04 ^Ad^	13.99 ± 0.43 ^Ad^
C1	77.41 ± 1.20 ^Ab^	0.92 ± 0.03 ^Ac^	15.43 ± 0.27 ^Ac^
C2	77.55 ± 0.59 ^Ab^	1.73 ± 0.18 ^Aa^	17.49 ± 0.36 ^Ab^
C3	79.14 ± 1.02 ^Aa^	1.51 ± 0.22 ^Ab^	19.09 ± 0.61 ^Aa^
C4	79.62 ± 0.60 ^Aa^	1.00 ± 0.016 ^Ac^	19.31 ± 0.21 ^Aa^
blank	78.36 ± 1.54 ^Aa^	0.50 ± 0.04 ^Ae^	13.99 ± 0.43 ^Ae^
E1	68.72 ± 0.82 ^Bb^	18.63 ± 0.51 ^Bd^	27.56 ± 0.89 ^Bd^
E2	64.59 ± 0.50 ^Bc^	22.52 ± 0.63 ^Bc^	30.15 ± 0.46 ^Bc^
E3	65.31 ± 0.29 ^Bc^	23.34 ± 0.67 ^Bb^	31.76 ± 0.48 ^Bb^
E4	64.92 ± 0.95 ^Bc^	24.71 ± 0.52 ^Ba^	36.50 ± 0.48 ^Ba^

Note: C1, C2, C3, and C4 were meat products, in which 25%, 50%,75%, and 100% fat was replaced by emulsions, respectively. E1, E2, E3, and E4 were meat products, in which 25%, 50%, 75%, and 100% fat was replaced by emulsions loaded with Lyc, respectively. ^a–e^ Different letters indicate significant differences within the same column (*p* < 0.05). ^A,B^ Different letters indicate significant differences between Lyc-loaded and non-loaded samples at the same substitution level. Values are expressed as mean ± standard deviation (SD).

**Table 4:** Textural properties of the meat with different proportions of emulsions replacing fat.

	Hardness (N)	Resilience (ratio)	Cohesiveness (ratio)	Springiness (mm)	Chewiness (mj)
blank	2.25 ± 0.09 ^Ae^	0.12 ± 0.04 ^Aa^	0.66 ± 0.03 ^Ab^	0.86 ± 0.03 ^Aa^	1.05 ± 0.03 ^Ad^
C1	2.54 ± 0.03 ^Ad^	0.12 ± 0.01 ^Aa^	0.65 ± 0.02 ^Abc^	0.86 ± 0.04 ^Aa^	1.35 ± 0.05 ^Ac^
C2	2.88 ± 0.04 ^Ac^	0.12 ± 0.04 ^Aa^	0.65 ± 0.02 ^Abc^	0.88 ± 0.05 ^Aa^	1.44 ± 0.34 ^Abc^
C3	3.17 ± 0.06 ^Ab^	0.10 ± 0.04 ^Aa^	0.69 ± 0.01 ^Aa^	0.86 ± 0.06 ^Aa^	1.61 ± 0.06 ^Aab^
C4	3.43 ± 0.04 ^Aa^	0.13 ± 0.03 ^Aa^	0.63 ± 0.01 ^Ac^	0.88 ± 0.04 ^Aa^	1.72 ± 0.04 ^Aa^
blank	2.25 ± 0.09 ^Ae^	0.12 ± 0.04 ^Aa^	0.66 ± 0.03 ^Aab^	0.86 ± 0.03 ^Aa^	1.05 ± 0.03 ^Ab^
E1	2.80 ± 0.02 ^Bd^	0.11 ± 0.02 ^Aa^	0.64 ± 0.02 ^Ab^	0.87 ± 0.03 ^Aa^	1.61 ± 0.04 ^Ba^
E2	3.07 ± 0.04 ^Bc^	0.10 ± 0.02 ^Aa^	0.66 ± 0.02 ^Aab^	0.88 ± 0.02 ^Aa^	1.68 ± 0.28 ^Aa^
E3	3.27 ± 0.03 ^Bb^	0.12 ± 0.03 ^Aa^	0.66 ± 0.03 ^Bab^	0.88 ± 0.02 ^Aa^	1.79 ± 0.27 ^Ba^
E4	3.50 ± 0.04 ^Ba^	0.10 ± 0.03 ^Ba^	0.68 ± 0.03 ^Ba^	0.87 ± 0.07 ^Aa^	1.83 ± 0.52 ^Aa^

Note: C1, C2, C3, and C4 were meat products, in which 25%, 50%, 75%, and 100% fat was replaced by emulsions, respectively. E1, E2, E3, and E4 were meat products, in which 25%, 50%, 75%, and 100% fat was replaced by emulsions loaded with Lyc, respectively. ^a–e^ Different letters indicate significant differences within the same column (*p* < 0.05). ^A,B^ Different letters indicate significant differences between Lyc-loaded and non-loaded samples at the same substitution level. Values are expressed as mean ± standard deviation (SD).

## Data Availability

The original contributions presented in this study are included in the article. Further inquiries can be directed to the corresponding authors.
